# Intracholecystic papillary neoplasm in a pediatric patient: a case report

**DOI:** 10.1097/RC9.0000000000000233

**Published:** 2026-02-24

**Authors:** Eman Alhajj, Fatima MHD Rida Hajij, Mohammad Rashed Al Abdalla, Soumar Mueen Alziadan, Alaa Aldirani, Ahmad Jamil Abbas

**Affiliations:** a Department of General Surgery, Damascus Hospital, Damascus, Syrian Arab Republic; b Department of Gastroenterology, Damascus Hospital, Damascus, Syrian Arab Republic

**Keywords:** biliary tract neoplasms, case report, diagnostic challenge, gallbladder neoplasms, intracholecystic papillary neoplasm, pediatric, whipple procedure

## Abstract

**Introduction:**

Intracholecystic papillary neoplasm (ICPN) is a rare premalignant tumor of the biliary tract, almost exclusively reported in adults.

**Case Presentation:**

A 14-year-old female with a history of cholelithiasis presented with jaundice, right upper quadrant pain, and weight loss. Imaging revealed a massively dilated common bile duct containing a tissue mass. Endoscopic retrograde cholangiopancreatography (ERCP) with biopsy was pivotal, confirming an intraductal papillary neoplasm. Due to refractory obstruction and intraoperative discovery of duodenal invasion, the surgical plan was altered to a pancreaticoduodenectomy (Whipple procedure). Histopathology confirmed ICPN with moderate dysplasia. The patient was discharged but succumbed to massive gastrointestinal hemorrhage 1-month postoperatively.

**Clinical Discussion:**

This case expands the known demographic for ICPN to include pediatric patients. The diagnostic challenge is highlighted, as ICPN’s radiological features often mimic other biliary malignancies, a difficulty compounded in this case by the unavailability of magnetic resonance cholangiopancreatography. ERCP with biopsy was essential for correct preoperative diagnosis. Despite successful macroscopic resection, the tumor exhibited aggressive local invasion, and the patient suffered a fatal complication, underscoring the high morbidity of major hepatobiliary surgery.

**Conclusion:**

This case underscores the exceptional rarity of ICPN in the pediatric population and highlights significant diagnostic challenges. The initial misdiagnosis, partly due to limitations in obtaining advanced imaging, emphasizes that ICPN can mimic other biliary malignancies. A high index of suspicion and the utilization of ERCP with biopsy are crucial for preoperative diagnosis. This report expands the known demographic for this disease and illustrates its potential for aggressive clinical behavior.

## Introduction

Intracholecystic papillary neoplasm (ICPN) is a rare, premalignant biliary tract tumor found predominantly in the gallbladder and rarely in the common bile duct. It typically affects adults, with a female predominance and a reported age range of 38–83 years^[^1^]^. While often asymptomatic, it can present with right upper quadrant pain or jaundice due to biliary obstruction^[^2^]^, with a definitive diagnosis relying on histopathological examination^[^3^]^. This report describes the first documented case of ICPN in a patient under 18 years of age. We report a fatal case of a 14-year-old female with ICPN – the first documented instance in a patient under 18 years of age – who unfortunately succumbed to postoperative complications 1 month after undergoing a Whipple procedure.

HIGHLIGHTS

- Maintain a high index of suspicion for ICPN in pediatric patients with biliary obstruction, as it can mimic more common malignancies and timely diagnosis is crucial.
- ERCP with biopsy is the definitive diagnostic tool when advanced imaging is unavailable, and surgeons must be prepared for extensive resection due to the potential for locally aggressive disease, despite only moderate dysplasia on histology.

This case report has been reported in line with the SCARE checklist.^[^4^]^.

## Case presentation

We present a 14-year-old Arab female presented with a 4-week history of intermittent right hypochondrial pain, radiating to the right flank. The pain was exacerbated by oral intake and was unrelated to defecation. Associated symptoms included jaundice (noted in the skin, sclera, and conjunctiva), dark urine, unquantified fever, anorexia, and weight loss. Her medical history was significant for cholelithiasis diagnosed at age 9.

On examination, the patient was icteric. Initial laboratory studies revealed leukocytosis (WBC 20,700/µL), anemia (Hb 8.1 g/dL), and significant cholestasis with elevated total bilirubin (4.9 mg/dL) and alkaline phosphatase (1060 U/L).

The diagnostic workup began with an abdominal ultrasound, which revealed a thickened gallbladder wall containing a highly echogenic component suspicious for gallstones. A cystic structure connecting to the bile ducts was identified, accompanied by significant dilation of both the intra- and extra-hepatic biliary systems, most pronounced on the left. The common bile duct was markedly dilated to 22 mm and contained highly echogenic components that lacked acoustic shadowing. Subsequently, an endoscopic retrograde cholangiopancreatography (ERCP) was performed. Following a sphincterotomy, cannulation and opacification of the CBD confirmed severe fusiform dilation with an intraluminal filling defect. A biopsy was taken from the visualized intraductal polypoid tissue, and a 9 cm, 10-French biliary stent was placed.

**Figure 1.**
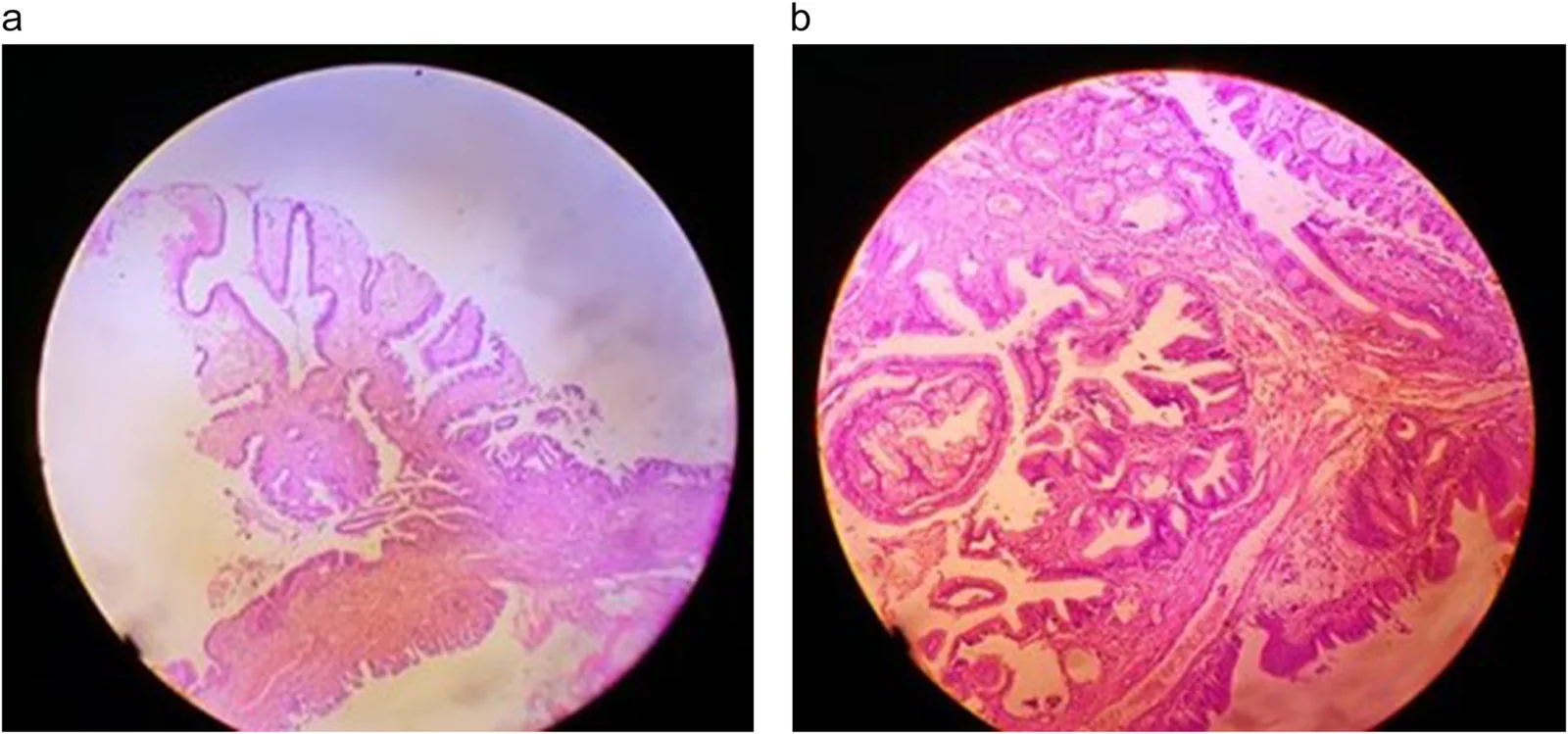
(a) Proliferation composed of papillary structures lined by epithelial cells showing intestinal and gastric pyloric differentiation. (b) Focus of neoplasm composed of back to back tubular glands surrounded to fibrotic stroma and lined by cells revealing gastric pyloric metaplasia with moderate dysplasia.

**Figure 2.**
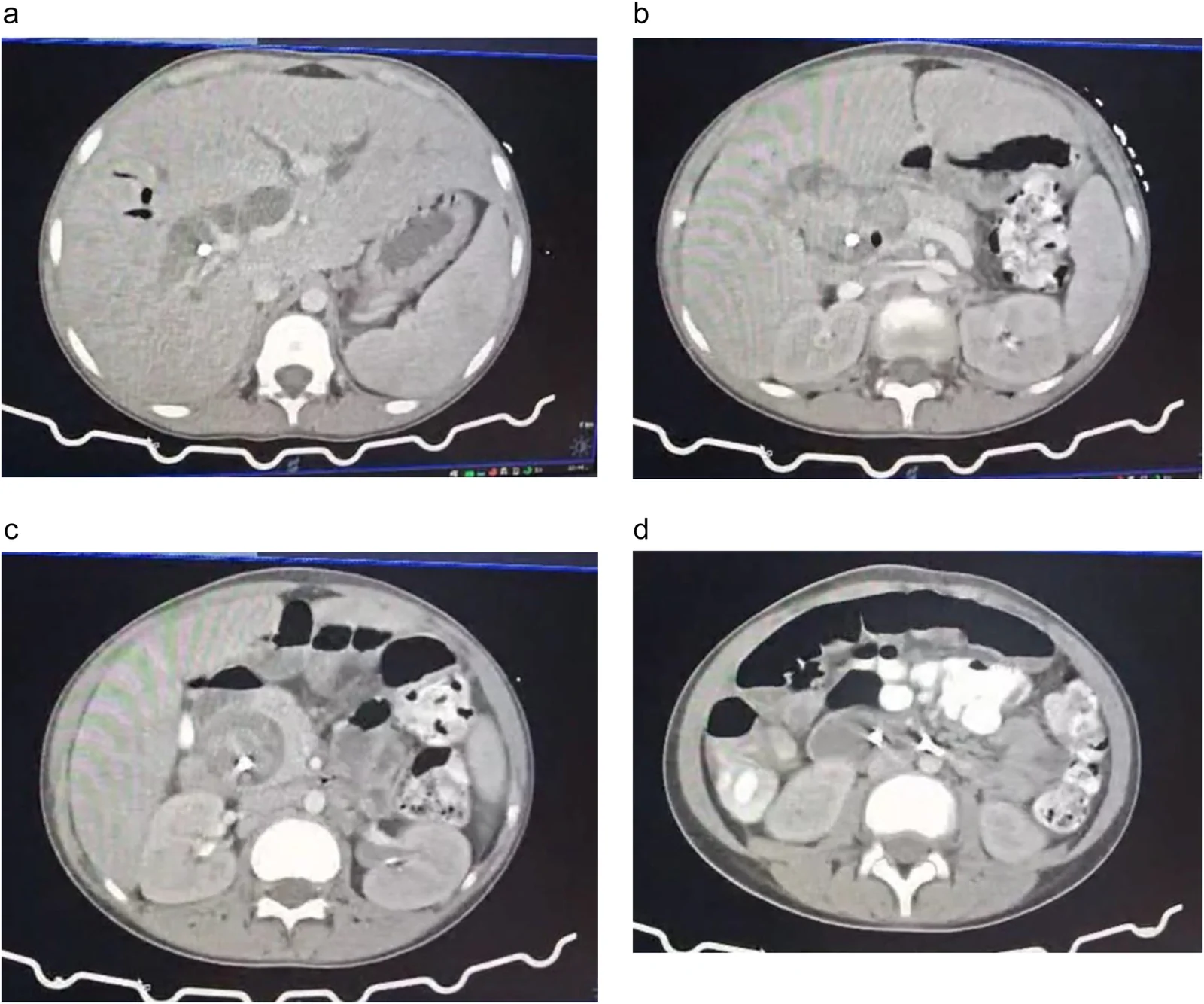
(a) Dilatation of the common bile duct with an intraductal stone. (b) Heterogeneous mass with calcification at the head of the pancreas. (c) Well-defined mass with calcification at the head of the pancreas. (d): Calcification at the distal end of the common bile duct.

The ERCP findings were consistent with a type IB choledochal cyst containing a tissue mass, and the subsequent pathology report confirmed the diagnosis of an intraductal papillary neoplasm of the biliary tract (Fig. 1). Further evaluation with a contrast-enhanced abdominal CT scan corroborated the severe biliary dilation, showing the common bile duct reaching a maximum diameter of 4 cm. The scan identified an enhancing tissue mass measuring 3 × 5 × 7 cm within the dilated duct without clear invasion of adjacent structures. Additionally, a few hypodense hepatic foci were noted, the largest measuring 6 mm in segment VIII, which raised suspicion for metastatic deposits. The overall radiological impression was that of a Klatskin tumor with suspected secondary hepatic deposits (Fig. 2).

Due to persistent obstructive jaundice refractory to conservative management, the patient was taken for exploratory surgery. A bilateral subcostal incision was made. The critical intraoperative finding was tumor extension into the duodenal wall. This local invasion necessitated a radical change in the surgical strategy; the planned choledochojejunostomy was abandoned in favor of a pancreaticoduodenectomy (Whipple procedure) to achieve a complete (R0) resection.

The resected specimen was submitted for pathological analysis. The final histopathology confirmed an intraductal/intracholecystic papillary and tubular neoplasm with moderate dysplasia. The patient’s initial postoperative recovery was uneventful, and she was discharged in stable condition. Approximately 1 month later, she represented with multiple episodes of gastrointestinal hemorrhage. Despite intensive medical and endoscopic interventions, her clinical status declined, and she succumbed to her condition.

### Timeline of key events

- **Week 1–4:** Symptom onset (pain, jaundice, weight loss).
- **Initial Presentation**: Clinical examination and laboratory studies revealing leukocytosis, anemia, and cholestasis.
- **Diagnostic Work-up**: Abdominal ultrasound followed by ERCP with biopsy and biliary stenting, then contrast-enhanced CT.
- **Week 5:** Exploratory surgery with intraoperative discovery of duodenal invasion, leading to a pancreaticoduodenectomy (Whipple procedure).
- **Post-operative Month**: Initial stable recovery and discharge.
- **One-Month Post-op**: Representation with massive gastrointestinal hemorrhage leading to mortality.

## Discussion

This report describes the first documented and tragic case of intracholecystic papillary neoplasm (ICPN) in a pediatric patient, a finding that dramatically expands the clinical and demographic spectrum of this rare biliary tract tumor. ICPN, recognized as the gallbladder counterpart to intraductal papillary neoplasm of the bile duct (IPNB), is characterized by pre-invasive, grossly visible papillary or tubulo-papillary growth within the gallbladder lumen^[^1,2^]^. While our case involved the common bile duct, the histopathological features are synonymous with the entity. Its occurrence is almost exclusively confined to adults, with a peak incidence in the sixth and seventh decades of life and a notable female predominance^[^3,5^]^. The presentation of this pathology in a 14-year-old female is therefore exceptional and compels a re-evaluation of the differential diagnosis for pediatric biliary obstruction, particularly in patients with risk factors such as a history of cholelithiasis.

The diagnostic odyssey in this case underscores the profound challenge ICPN poses, primarily due to its ability to masquerade as more common biliary malignancies such as cholangiocarcinoma. Our initial radiological suspicion of a Klatskin tumor with hepatic metastases was a logical, albeit incorrect, interpretation based on the CT findings of an enhancing tissue mass within a massively dilated duct and associated hepatic hypodensities. This diagnostic pitfall is well-documented, as the imaging features of ICPN/IPNB – including ductal dilation and intraductal masses – often overlap with those of cholangiocarcinoma^[^6,7^]^. This challenge was significantly exacerbated by the unavailability of magnetic resonance cholangiopancreatography (MRCP) in our setting. This challenge was exacerbated by significant limitations in our diagnostic workup. While ultrasound and CT were performed, MRCP, the noninvasive cornerstone for delineating biliary anatomy and characterizing intraductal lesions^[^8,9^]^, was unavailable. This highlights a critical constraint in resource-limited settings and likely contributed to the initial misdiagnosis.

In this context, ERCP with biopsy proved to be the pivotal diagnostic modality. ERCP allowed for direct visualization, therapeutic stenting, and, most importantly, tissue acquisition. The biopsy, which revealed an intraductal papillary neoplasm, was crucial in steering the diagnosis away from a conventional cholangiocarcinoma. This underscores the indispensable role of histopathological sampling in achieving a preoperative diagnosis when imaging is equivocal or advanced modalities are inaccessible^[^10^]^.

The management of ICPN is fundamentally surgical, with the goal of complete resection, as the lesion carries a significant risk of harboring or progressing to invasive carcinoma^[^11,12^]^. The intraoperative discovery of duodenal invasion was a critical turning point, revealing a degree of local aggressiveness that is highly unusual for a lesion with only moderate dysplasia on final pathology. This finding necessitated a radical shift from a planned choledochojejunostomy to a pancreaticoduodenectomy (Whipple procedure). It directly justified the radical shift from the planned, more limited choledochojejunostomy to an extensive pancreaticoduodenectomy (Whipple procedure) to ensure complete tumor removal. While ICPN is generally associated with a more favorable prognosis than conventional gallbladder cancer^[^13^]^, our case demonstrates that it can exhibit locally invasive behavior, demanding extensive resection for potential cure.

Despite a macroscopically successful and technically optimal surgical resection, the patient’s outcome was tragic. Her death from massive gastrointestinal hemorrhage 1 month postoperatively is a stark reminder of the profound morbidity and mortality associated with major hepatobiliary-pancreatic surgery. Potential etiologies for such a catastrophic hemorrhage include pseudoaneurysm formation (e.g., of the gastroduodenal artery), anastomotic ulceration, or stress-related mucosal injury. This devastating outcome underscores that even when a rare and complex diagnosis is correctly made and addressed with definitive surgery, the procedure itself carries life-threatening risks, particularly in young, potentially malnourished patients. It argues for meticulous surgical technique and extremely vigilant postoperative monitoring in such high-stakes scenarios.

This case has several limitations. It is a single report from a single institution, and the unavailability of MRCP and genetic/molecular profiling of the tumor are notable shortcomings. The absence of such data leaves unanswered the question of whether pediatric ICPN possesses distinct molecular drivers that explain its apparent aggressive clinical behavior in this instance. Future research should focus on the molecular characterization of such rare tumors to better understand their pathogenesis and identify potential therapeutic targets.

## Conclusion

This case represents the first documented occurrence of Intracholecystic Papillary Neoplasm (ICPN) in a pediatric patient. It highlights the significant diagnostic challenge this tumor presents, as it can mimic other biliary malignancies, leading to initial misdiagnosis. The definitive role of histopathology in achieving the correct diagnosis is underscored. The finding of duodenal invasion, despite moderate dysplasia, illustrates the potential for locally aggressive behavior and necessitated a radical surgical approach (Whipple procedure). Despite successful surgical resection with a Whipple procedure, the patient’s fatal outcome illustrates the potential for an aggressive disease course and underscores the need for awareness of this rare entity in all age groups.
